# P-1097. Educative Effectiveness of Fully Immersive 360° Virtual Reality System on Hand Hygiene Practice: A Pilot Randomized Controlled Trial

**DOI:** 10.1093/ofid/ofaf695.1292

**Published:** 2026-01-11

**Authors:** Hideharu Hagiya, Mahiro Izumi, Akio Gofuku

**Affiliations:** Okayama University Hospital, Okayama, Okayama, Japan; Okayama University, Okayama, Okayama, Japan; Okayama University, Okayama, Okayama, Japan

## Abstract

**Background:**

Educational impact of virtual reality (VR) technology for healthcare practitioners has been increasingly acknowledged, including training aids for infection prevention and control (IPC). We developed a VR education system and evaluated its clinical utility for promoting hand hygiene practices.

**Methods:**

This is a prospective, two-week, randomized-control study conducted at Okayama University Hospital, Japan, from November 2023 to January 2024. The fully immersive 360° VR system (VIVE Pro Eye), using a head-mounted display and sensing gloves, was applied to develop three healthcare tasks in a virtual patient room. We monitored the baseline usage data of portable hand-rubbing alcohol of all the participants in the first week, and subsequently randomized participants into 1:1 groups (VR training group and video lecture group). The primary outcome was the differences in the consumption amount of the hand-rubbing alcohol before and after the interventions.

**Results:**

A total of 22 participants (18 medical students and 4 residents) were recruited. Before the intervention, alcohol usage was not significantly different between the VR training and video lecture groups (8.2 g vs. 5.3 g). After the intervention, a significant increase in the use of hand-rubbing alcohol was observed in the VR training group (8.2 g vs 16.2 g; *p*=0.019), whereas there was no significant difference in the video lecture group (5.3 g vs 7.5 g; *p*=0.83)

**Conclusion:**

Hand hygiene adherence in healthcare settings can be improved through the introduction of VR-based education owing to its immersive learning capabilities, interactive involvement, and affordability in experiencing complicated scenarios.

**Disclosures:**

All Authors: No reported disclosures

